# Super resolution-based methodology for self-supervised segmentation of microscopy images

**DOI:** 10.3389/fmicb.2024.1255850

**Published:** 2024-03-12

**Authors:** Vidya Bommanapally, Dilanga Abeyrathna, Parvathi Chundi, Mahadevan Subramaniam

**Affiliations:** Department of Computer Science, University of Nebraska, Omaha, NE, United States

**Keywords:** super-resolution, image segmentation, self-supervised learning, microscopy images, image resolution

## Abstract

Data-driven Artificial Intelligence (AI)/Machine learning (ML) image analysis approaches have gained a lot of momentum in analyzing microscopy images in bioengineering, biotechnology, and medicine. The success of these approaches crucially relies on the availability of high-quality microscopy images, which is often a challenge due to the diverse experimental conditions and modes under which these images are obtained. In this study, we propose the use of recent ML-based image super-resolution (SR) techniques for improving the image quality of microscopy images, incorporating them into multiple ML-based image analysis tasks, and describing a comprehensive study, investigating the impact of SR techniques on the segmentation of microscopy images. The impacts of four Generative Adversarial Network (GAN)- and transformer-based SR techniques on microscopy image quality are measured using three well-established quality metrics. These SR techniques are incorporated into multiple deep network pipelines using supervised, contrastive, and non-contrastive self-supervised methods to semantically segment microscopy images from multiple datasets. Our results show that the image quality of microscopy images has a direct influence on the ML model performance and that both supervised and self-supervised network pipelines using SR images perform better by 2%–6% in comparison to baselines, not using SR. Based on our experiments, we also establish that the image quality improvement threshold range [20–64] for the complemented Perception-based Image Quality Evaluator(PIQE) metric can be used as a pre-condition by domain experts to incorporate SR techniques to significantly improve segmentation performance. A plug-and-play software platform developed to integrate SR techniques with various deep networks using supervised and self-supervised learning methods is also presented.

## 1 Introduction

Microscopy images play a major role in analyzing and predicting the behavior of complex systems in various fields, such as systems, bioengineering, living materials, and medicine (Sommer and Gerlich, 2013; Xing and Yang, 2016; Min et al., 2017). Computational analyses of these images using traditional image analysis algorithms is a non-trivial task as these images are captured under diverse experimental conditions and are widely varied in image quality in terms of noise, blur, and magnification (Bulgarevich et al., 2018; Ma et al., 2023). Machine learning (ML) techniques using deep neural networks (DNNs) have begun to surpass traditional image analysis methods in analyzing image datasets of varying quality and characteristics. However, usually, images have to be manually annotated by experts to build ML methods, interpreting whose outputs again involve a careful study of the images. Computational approaches that can improve the quality of microscopy datasets and the integration of these approaches into image analysis pipelines of DNNs are essential for effective analyses of microscopy images using ML techniques. Super-resolution (SR) is a set of well-established image transformation algorithms that have been used to produce a high-resolution (HR) image from a given low-resolution (LR) image (Park et al., 2003). Recently, several sophisticated ML methods based on DNNs and transformers have been designed and become the de facto approaches for successfully performing SR itself. However, the use of such ML-based SR approaches and their integration into the DNN pipelines used to analyze microscopy pipelines is relatively new and deserves more attention.

This study presents a detailed analysis of several ML-based SR approaches and their integration into a variety of DNN pipelines using supervised learning (SL) and multiple self-supervised learning (SSL) approaches for performing the segmentation of microscopy image datasets. We consider microscopy datasets of varying image quality quantitatively measured using multiple quality metrics, study, and establish all ML-based SR approaches to improve image quality though at different levels. Pipelines for the segmentation of various objects in microscopy images are designed, incorporating ML-based SR approaches, and it is established that the segmentation performance of the pipelines incorporating the ML-based SR approaches is higher than the pipelines, where SR is not employed. We also show that the quality of microscopy images has a significant impact on the segmentation performance of both SL and SSL methods. Our results in this study highlight the benefits of using ML-based SR approaches for the segmentation of microscopy images and provide image quality-based criteria for the selection of an appropriate ML-based SR approach for various ML approaches. Given the important role of ML-based SR approaches, we also develop and present a software platform that allows domain experts where they can perform the segmentation of microscopy images using a variety of ML-based SR methods integrated with SL and SSL approaches.

Our main contributions in this study include

- Integrating deep learning-based SR methods with SL and SSL methods to improve semantic segmentation of microscopy images of varying quality and volumes.
- The use of image quality metrics to predict the effectiveness of applicability of the SR methods for semantic segmentation of microscopy images.
- Quality threshold value ranges which, when satisfied by the output images of several well-established SR methods, lead to improved segmentation performance across diverse microscopy images.
- In addition to providing guidelines to domain experts regarding the use of SR methods in their applications, these threshold value ranges and also opens up the possibility of using SR for data augmentation for SSL methods.
- A pipeline where the use of ML-based SR methods leads to improvements in image segmentation performed across a variety of ML techniques including SL and a variety of SSL methods.
- A software workbench incorporating state-of-the-art ML-based SR methods, SL, and contrastive and non-contrastive SSL methods that can be used by domain experts to segment microscopy images collected under different experimental conditions.

To the best of our knowledge, our work is novel in combining SR techniques with SSL for microscopy images and provides quantitative pre-conditions for using these techniques. Data augmentation is a key approach in SSL to address low data volume microscopy images. This work suggests the use of *conditional data augmentation*, where data augmentation such as super-resolution is applied only when certain thresholds are satisfied. This study highlights the potential benefits of ML-based SR approaches for image analyses, especially for microscopy images that are collected under diverse experimental conditions. Our focus in this study is on the particular image analysis task of segmentation. However, ML-based SR approaches quantitatively improve image quality and are likely to improve the accuracy of a wide variety of image analysis tasks, including classification, object detection, and instance segmentation, and hence are an important tool in the bioengineering and medical computational toolkit.

## 2 Related work

### 2.1 Segmentation using SSL

Electron microscopy plays a critical role in microscale biomaterial characterization by providing sub-nanometer structural resolution and uncovering hidden topological and compositional features (Vladr et al., 2009). As a result, electron microscopy finds extensive application in diverse fields including material science, chemistry, nanomedicine, physics, and beyond (De Haan et al., 2019; Perez et al., 2022). Segmentation, also known as pixel-level classification, is employed to identify objects in microscopy images. Different architectural techniques such as single-staged (Long et al., 2015; Ronneberger et al., 2015) and two-staged (He et al., 2017) architectures can effectively perform microscopic image segmentation by training models using SL techniques (Vuola et al., 2019; Abeyrathna et al., 2021, 2022b; Kromp et al., 2021). Typically, training high-performance DNN using SL requires large amounts of manually annotated data (Huang et al., 2019). Alternatively, SSL techniques (Wang et al., 2018; Liang et al., 2021; Zhang et al., 2021; Chen et al., 2023) can be employed to build models that learn representations from unlabeled data and then fine-tune the model using limited amounts of manually annotated data.

Numerous applications (Bai et al., 2019; Chaitanya et al., 2020; Ouyang et al., 2020; Abeyrathna et al., 2022a) have leveraged SSL techniques for performing the segmentation of images. Deep learning is well-known and has been used in previous biomedical studies (Kha et al., 2022; Yuan et al., 2023). For example, SSL-based segmentation applications on biomedical microscopic images were highly adopted (Shurrab and Duwairi, 2022; Rettenberger et al., 2023; Sánchez et al., 2023). However, gathering sufficient volumes of annotated microscopy images is a tedious and time-consuming task that requires considerable domain expertise. Training with scarce annotated data leads to the *overfitting* problem (models that perform well on the data on which they are trained but not on others) (Hesamian et al., 2019), which makes the models unusable. Several approaches including data augmentation, dividing each image into multiple patches (Dou et al., 2017), and alternative ML approaches such as active learning (Chakravarthy et al., 2022) have been used to reduce the manual annotation effort. While many of these methods generally enhance the performance of deep learning models, not all techniques are suitable for analyzing microscopy image datasets that often consist of images of wide-ranging resolutions and magnifications. Without sharp and consistent identifications of the objects of interest across microscopy image datasets, the application of data augmentation, patching, and active learning often leads to a class imbalance in the output image data and lowers the accuracy of the resulting models (Dou et al., 2017; Abdollahi et al., 2020).

To address these problems and produce high-performing deep learning models for the segmentation of microscopy image data, we propose the use of image SR techniques. We design deep ML pipelines for microscopy image segmentation incorporating SR techniques. By applying SR, we obtain high-quality image patches, which ultimately enhance the performance of deep learning models on image segmentation.

### 2.2 SR methods

SR is a classic, low-level computer vision task, which has recently drawn significant attention due to its applicability in various image analysis applications. This task aims to reconstruct the HR version of the LR image. SR approaches can be mainly categorized into interpolation-based methods, reconstruction-based methods, and learning-based methods (Yang et al., 2019). These methods analyze the available image data to extract high-frequency details, which are then utilized to enhance an LR image. By leveraging these details, these algorithms improve the sharpness and level of detail in the final output, resulting in an HR image.

Typically, lower resolutions in images are attributed to image degradation, occurring due to scaling or the introduction of noise in images. Many approaches use simple degradation functions, such as a single downsampling operation to model image degradation and try to invert these functions using SR methods to generate HR counterparts. In fact, numerous datasets for SR are constructed based on this assumption, with bicubic interpolation accompanied by anti-aliasing being the most commonly used downsampling technique. It is important to note that real-world degradation can be influenced by various factors, including compression artifacts, anisotropic degradations, sensor noise, and speckle noise. Nonetheless, researchers strive to approximate the degradation functions and reverse their effects to enhance the LR images and recover plausible HR counterparts (Wang et al., 2020).

DNN-based approaches have been the de facto methodology for applying SR to generate HR images. The SRCNN by Dong et al. (2014) can be recognized as the first deep-learning-based approach applying SR. SRCNN is an approach that directly learns the mapping between LR and HR images. This is achieved through a deep CNN architecture. Some of the other techniques that use CNN in an end-to-end manner to apply SR (Kim et al., 2016a,b; Lim et al., 2017). Deep Recursive Convolutional Network (DRCN) (Kim et al., 2016b) introduces recursive learning, where the same modules are applied multiple times in a recursive manner, to learn higher level features. VDSR (Kim et al., 2016a) is a single network that is capable of jointly handling SR for multiple scales. EDSR (Lim et al., 2017) focuses on a specific SR scale, while a multi-scale architecture reconstructs various scales of HR images within a single model.

#### 2.2.1 Generative adversarial network (GAN)-based SR

Due to the successes achieved by GAN-based techniques in generating images, GAN-based architectures are extensively used in SR tasks. Super-resolution Generative Adversarial Network (SRGAN) (Ledig et al., 2017) is one of the GAN-based SR techniques. SRGAN utilizes a perceptual loss function, consisting of an adversarial loss and a content loss, to generate HR images. Enhanced Super-Resolution Generative Adversarial Network (ESRGAN) (Wang et al., 2018) and Blind Super-Resolution Generative Adversarial Network (BSRGAN) (Zhang et al., 2021) are improvements over SRGAN, which are state-of-the-art high-performing GAN-based SR techniques.

The ESRGAN model's architecture incorporates multiple Residual Dense Blocks without batch normalization layers. Training is optimized through techniques, such as residual scaling and smaller initialization. To enhance texture recovery, a relativistic GAN is employed as the discriminator, enabling the generator to discern image realism. The perceptual loss is further improved by utilizing features before activation, resulting in more accurate brightness and realistic textures. ESRGAN was trained on DIV2K and Flickr2K datasets. ESRGAN is trained on Adam optimizer with a learning rate initialized to 1 × 10^−^4 on mini-batches of size 16.

The BSRGAN employs a new degradation model to overcome the limitations of existing models in capturing diverse degradation processes in real images. The proposed model combines shuffled blur, downsampling, and noise degradations, utilizing Gaussian kernels, various downsampling methods, and multiple noise sources, where LR images are obtained without any prior knowledge about the original HR images. BSRGAN incorporates a perceptual loss function similar to ESRGAN to improve image quality and also utilizes an attention mechanism to mitigate the misalignment artifacts between the LR and HR images. Training a deep blind ESRGAN super-resolver with this model enables the enhancement of both synthetic and real images with different degradations. BSRGAN was trained on a wider variety of datasets including DIV2K, Flick2K, and WED datasets and 2,000 face images from FFHQ dataset. BSRGAN also uses Adam with a fixed learning rate of 1 × 10^−^5 over a batch size of 48. Experimental results highlight a significant improvement in the practicality of deep super-resolvers, offering a powerful solution for real-world single-image SR applications.

#### 2.2.2 Transformer-based SR techniques

Recently, SR approaches have been developed using transformer-based architectures (Vaswani et al., 2017), to address the limitations of CNN-based architectures in handling content-dependent interactions and modeling long-range dependencies (Cao et al., 2021; Chen et al., 2021). Swin Transformer (Liu et al., 2021) integrates CNN and transformer to produce SwinIR platform (Liang et al., 2021) for image restoration. SwinIR comprises shallow and deep feature extraction modules, utilizing convolution and residual Swin Transformer blocks. Due to attention mechanisms, SwinIR captures the relations between image patches, leading to considerably better results than CNN-based approaches. The SwinIR architecture consists of three main modules, namely, shallow feature extraction, deep feature extraction, and high-quality image reconstruction. However, Swin Transformer's shift window mechanism has limitations in cross-window information interaction and exploits less number of pixels in the input image compared with the CNN-based SR approaches. To address these limitations and leverage the transformer's potential for SR, the Hybrid Attention Transformer (HAT) (Chen et al., 2023) is introduced. HAT combines channel attention and self-attention, harnessing global information utilization and powerful representation. An overlapping cross-attention module facilitates direct interaction among adjacent window features. HAT activates more pixels for reconstruction, leading to improved SR performance. SwinIR was trained on DIV2K dataset and HAT was trained on DIV2K and Flickr2K datasets.

### 2.3 Our contribution

To improve image resolution of SEM images, the use of deep learning networks is considered by authors in De Haan et al. (2019). They propose a deep-learning approach to enhance the lateral resolution of SEM images. They train a CNN using co-registered HR and LR SEM images to improve individual image quality without additional sample preparation. By utilizing an experimentally acquired training dataset, the network accounts for aberrations and noise in the imaging system. This data-driven approach reduces electron beam scanning time, enabling lower magnification scans over larger fields of view while maintaining image quality and reducing sample charging and beam damage. In our earlier studies, Abeyrathna et al. (2022a) used the BSRGAN technique along with SSL methods for classifying microscopy images of a biofilm dataset. Ashaduzzman et al. (2022) showed how images in that dataset can be effectively segmented after pre-processing the images with BSRGAN, ESRGAN, and SwinIR methods.

This study conducts a comprehensive study of the role and effectiveness of various SR techniques and their impacts on segmenting microscopy images across multiple datasets by using SL and SSL approaches. We consider SL and multiple SSL methods and quantify the effectiveness of applying various SR methods in improving segmentation performance. We show that the image quality metrics can be improved using SR techniques and establish a good correlation between the quality and segmentation performance of the models built using SL and SSL approaches. The SR methods used here include BSRGAN, ESRGAN, SwinIR, and HAT. These methods were chosen from GAN and transformer SR techniques based on their high performance and wide usage. The SSL methods employed here include both contrastive and non-contrastive architectures. Our results empirically establish that SR techniques are an important tool and should be an intrinsic part of any deep learning pipeline that is used for the segmentation of microscopy images.

## 3 Materials and methods

The overall study provides a comparative overview of different SL and SSL deep learning architectures and the models trained on original microscopy images and those generated using the SR methods. The study makes the use of different microscopy image datasets processed with four state-of-the-art SR techniques to evaluate the segmentation performance of various SL and SSL models. Several traditionally used image quality metrics are used to assess the original and SR-generated images and correlate them with segmentation performance.

### 3.1 Datasets

**Figure 1:**
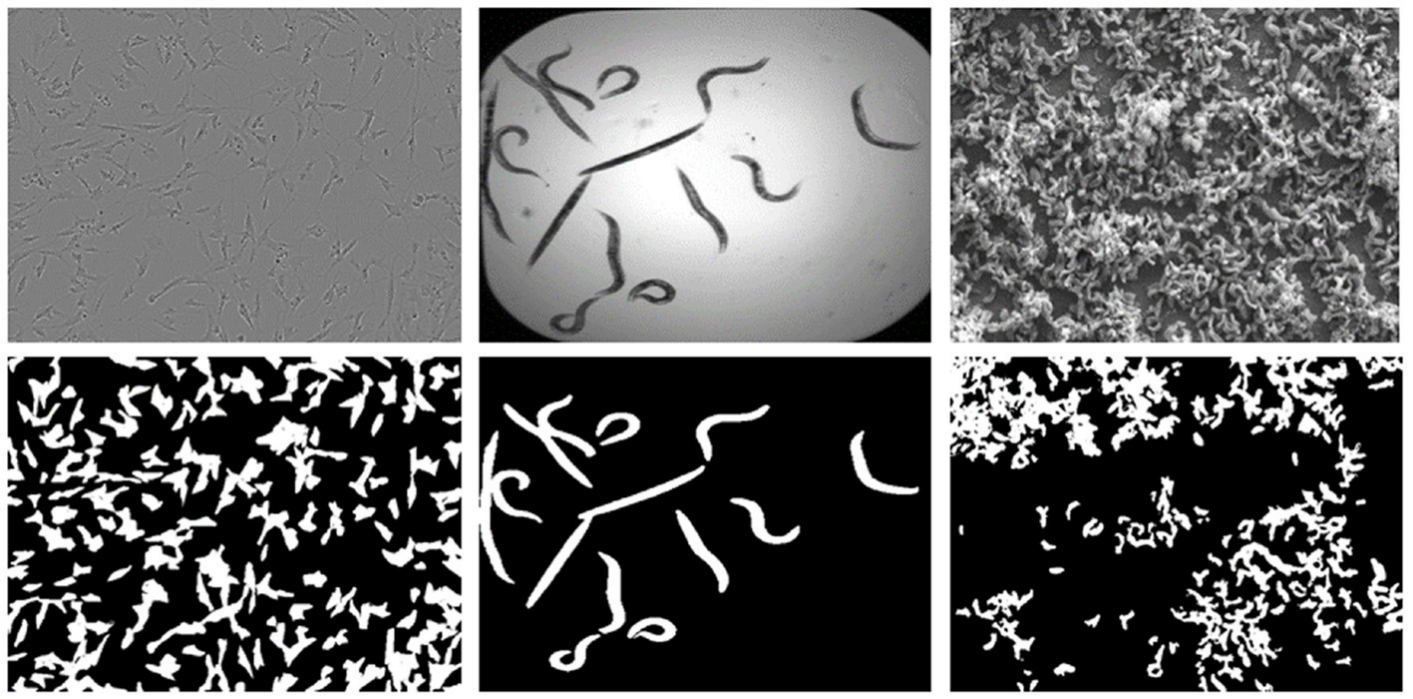
Sample images from each dataset showing the original images on the top and their corresponding Segmentation GT masks showing cells in the bottom, (from left) Livecells, *C. elegans*, and Biofilms.

The *LiveCell* dataset consists of 5,239 manually annotated and expert-validated microscopic images comprising various cell types (Edlund et al., 2021). In this study, we chose 155 images belonging to the cell type *SHSY5Y*, which are consistent with the other chosen datasets. These are gray-scaled images with a resolution of 704 × 520 px. The second dataset, *C.elegans* live/dead assay, Broad Bioimage Benchmark Collection (Accession number BBBC010, Version 1) (Ljosa et al., 2012), is a collection of publicly available microscopy images from a competition (Moy et al., 2009). This dataset consists of 100 images, and all the images are 16-bit grayscale, having a resolution of 696 × 520 px. The third *Biofilms* dataset is a private dataset consisting of a set of SEM images of a sulfate-reducing bacteria *Desulfovibrio alaskensis* (DA-G20) and their biofilms. The data were transformed into 7, 16-bit grayscale SEM images at a resolution of 1,024 × 758 px. More information regarding the data collection environments and measurements are discussed in the study by (Susarla et al., 2021; Abeyrathna et al., 2022a; Chakravarthy et al., 2022). Sample images from each of the datasets and their corresponding GT masks are shown in Figure 1. The selection of the above datasets was mainly based on the diversity of the morphological characteristics and imaging modalities, LiveCell belongs to Light microscopy, BBBC010 belongs to Bright field microscopy, and the Biofilm dataset belongs to SE microscopy imaging. Notably, the complexity of features to be learned in the BBBC010 dataset is comparatively lower for the segmentation task. However, machine learning approaches can exhibit more adaptability and flexibility for the segmentation task compared with deterministic approaches, which require various parameter changes for a given instance. The key idea is to show that even easily segmentable objects can benefit from controlled super-resolution improvements.

### 3.2 SSL techniques

**Figure 2:**
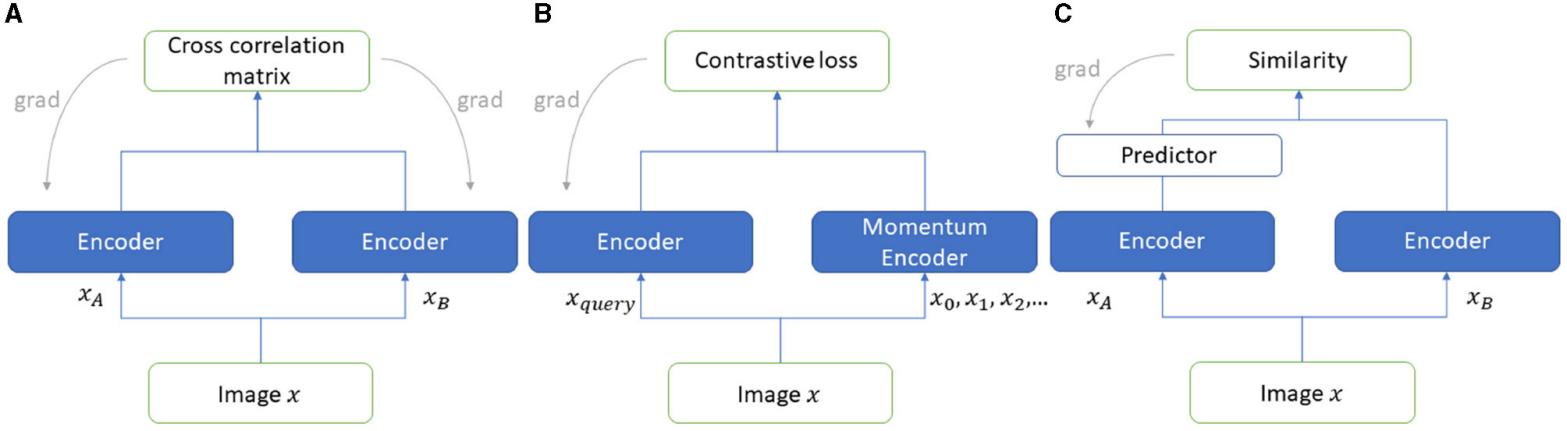
SSL frameworks used in the experiments **(A)** Barlow Twins, **(B)** MoCoV2, and **(C)** SimSiam.

We employed one contrastive and two non-contrastive SSL techniques to compare the efficiency of learning features from SR images. These state-of-the-art methods use augmentation as a pretext task for performing representation learning from unlabeled data. The three techniques are MocoV2, Barlow Twins, and Simsiam each of which is discussed in this section. The architectures are shown in Figure 2.

#### 3.2.1 MoCoV2

MoCoV2 (Chen et al., 2020) is one of the contrastive SSL learning approaches. Unlike non-contrastive approaches, this approach is based on instance discrimination, where positive and negative pairs of images are discriminated. The images belong to a positive set if they are augmented versions of the same image, otherwise images are paired as a negative set. There are two types of inputs to the MoCoV2 architecture, an encoded query and a set of encoded keys comprising keys that match the encoded query. The rest of the keys are termed negative keys for encoded queries. The contrastive loss function InfoNCE loss aims at reducing the distance between the matched pairs while increasing the distance between the negative samples by learning their similarities and dissimilarities, respectively. The architecture comprises two encoders, one encodes the query, and the other is called a momentum encoder which encodes and maintains a queue for the new keys dynamically (Figure 2B) (Abeyrathna et al., 2022a).

#### 3.2.2 Barlow twins

Barlow Twins (Zbontar et al., 2021) has been identified as one of the popular SSL frameworks, which utilizes two encoder models to extract feature representations and learn from unlabeled data. The two encoder network (referred to as “twins”) weights are adjusted during the training process, maximizing the similarity of two distorted (augmented) positive samples while attempting a cross-correlation matrix of the outputs of “twin” networks as close to the identity matrix as possible. The loss function which utilizes a cross-correlation matrix is known as “Barlow Twin loss”. The loss function consists of two parts, namely, (1) invariant term and (2) redundancy reduction term. The invariant term handles the noise factor of the data to enhance the robustness, whereas the redundancy reduction term ensures the invariance of the representations learned by the twin networks (See Figure 2C) (Abeyrathna et al., 2022a).

#### 3.2.3 SimSiam

SimSiam (Chen and He, 2021) is a state-of-the-art non-contrastive learning approach to SSL. This approach uses a Siamese twin network (see Figure 2A) with a stop gradient applied to one of the twins. This prevents the network from collapsing. Two different randomly chosen augmented versions are provided as inputs to the two network branches. A cosine similarity loss function is trained on the encoded features from each branch aiming to learn to maximize the similarity between them and thereby learning the invariant representations from the data. A predictor is applied on one of the network encoders here to enable the architecture to transform one of the augmented views and further compare this transformed view with the augmented view from the other network encoder. Different augmentations such as rotation, random horizontal/vertical flip, and Gaussian noise are randomly performed on the images.

### 3.3 Experiments

The experiments including the SSL and downstream model training and testing tasks were conducted on a LAMBDA QUAD Deep Learning Workstation with Intel(R) Core(TM) i9-9920X CPU (3.50GHz), with 24GB Nvidia Quadro RTX 6000 GPU and 128GB RAM.

**Figure 3:**
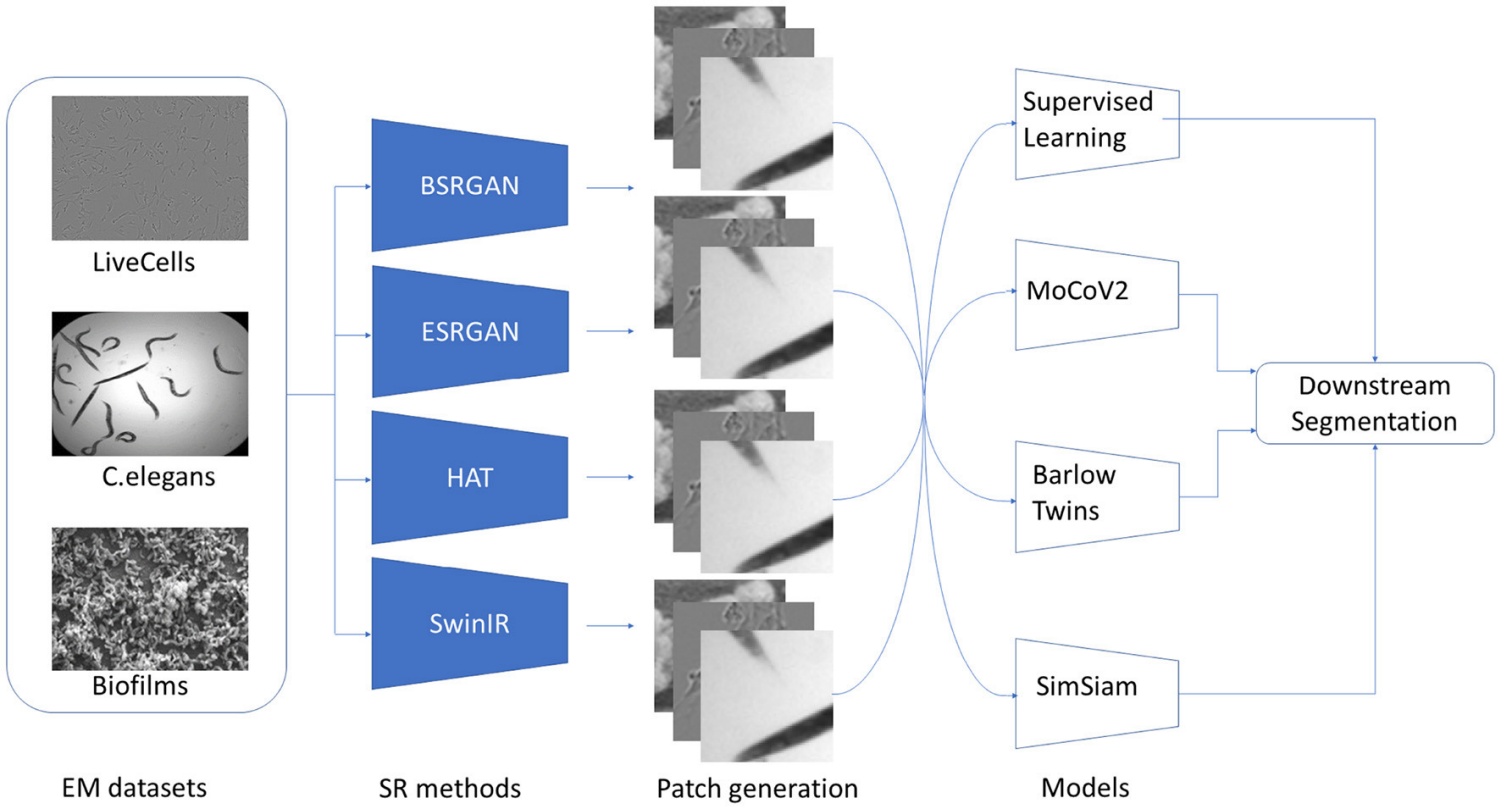
Stages in the experiments conducted beginning with datasets, the SR techniques, generation of patched datasets, SSL pre-training, and downstream segmentation.

The flow of the experiments is shown in Figure 3. The first step in the experiment process was to generate SR images for each of the three datasets - Biofilms, C.elegans, and LiveCells. As mentioned, four different SR methods BSRGAN, ESRGAN, HAT, and SwinIR were employed to generate images with resolutions × 2 and × 4 for each method. For generality, the original microscopy images and those output by SR methods with resolution × 2 were resized to their corresponding × 4 resolution sizes for each dataset. The original images were also resized to × 4 with bicubic interpolation to compare super-resolution and high-resolution segmentation performance. Consequently, we curated nine datasets for each of Biofilms, C.elegans, and LiveCells attributing to a total of 27 unique datasets. These nine datasets comprise an original dataset without SR and four different SR methods with two levels of scale (× 2 and × 4) generating eight combinations of datasets.

We randomly chose 80% of the images from each dataset for the SSL methods to learn representations without labels. The rest 20% of the data was labeled and used for fine-tuning the downstream task for SSL. To increase the volume of the datasets the images are patched. As the images contain microbial cells spatially distributed patching can capture cell information in each image patch. For the SSL pretext task, strided patching of the images was performed to generate patches with a size of 256 × 256. The patch size is determined from our previous experiments (Bommanapally et al., 2021; Abeyrathna et al., 2022a; Ashaduzzman et al., 2022) where we showed that increased patch size can often capture more relevant features of the objects in the biofilm microscopy images. The 27 datasets were patched to create approximately 66000 image patches for each dataset used for pre-training. The remaining 20% of the images were also patched with a patch size of 256 × 256 px for performing the downstream segmentation along with pixel-wise annotated masks for the cell class of the dataset. The 20% labeled data was further split into 70%, 20%, 10% train, validation, and test sets respectively. For SL, the entire dataset, which had labels, was used.

Following, three SSL frameworks MoCoV2, Barlow Twins, and SimSiam were trained on the patched datasets. The pre-training for all the frameworks used a batch size of 256 trained for 300 epochs. Augmentation was used as a pretext task for the three frameworks. Different augmentations were randomly applied to the patches including random crop, random color jitter, gaussian blur, and flip for all the SSL methods used. All the models use ResNet50 (He et al., 2016), as the backbone for the encoders. The trained weights were further used for the downstream tasks. The trained weights with ResNet50 were used as the backbone for the FCN ResNet50. The network is then fine-tuned with the patched dataset along with the ground truth (GT) masks. The model is trained for 100 epochs with a batch size of 16 and binary cross-entropy loss. Hyperparameters such as learning rate, momentum, and loss used for SSL were the same as the vanilla implementations of each model. For the finetuning of all the pretrained models, an adjustable learning rate was used with an initial learning rate set to 0.01, SGD optimizer with a momentum of 0.9 was used.

#### 3.3.1 Evaluation Metrics

##### 3.3.1.1 SR image quality

*Peak signal-to-noise ratio (PSNR)* is one of the widely used metrics to compare the image compression quality of an image over its reference (in our experiments reference image is the original image) image. We measured the PSNR values to understand the quality enhancement or the distortion that occurred due to the application of the SR methods. The metric calculates the ratio between the maximum possible power of a signal and the mean squared error (MSE). The formula for the PSNR metric is provided in Equation 1.


(1) $$\begin{array}{l} PSNR=10\times log_{10}\left(\frac{I^{2}}{MSE}\right) \end{array}$$

Here, *I* represents the maximum possible pixel value of an image, and the MSE value is calculated as the average of the squared differences between the corresponding pixels of the image and its reference image. A higher PSNR value indicates better image quality and a lower PSNR value indicates a higher amount of distortion due to the SR application. However, PSNR evaluates the quality of an image based on image pixel-level differences which is not the way the human observers perceived image quality.

*Structural Similarity Index (SSIM)* (Wang et al., 2004) is used as a metric to measure structural similarity between two images. SSIM is a widely used metric due to the fact that it is closer to the perceptual quality of images by the human visual system, which is often more sensitive to structural information rather than intensity or pixel-wise differences. SSIM metric mainly attempts to capture Luminance (*l*), Contrast (*c*), and Structure (*s*) when it compares an image to a reference image. The SSIM can be formulated as *SSIM* = *l*×*c*×*s*, where the three components *l*, *c*, and *s* are calculated as the mean, the variance, and the covariance of the corresponding image segments in the given and the reference image respectively.

*Perception-based Image Quality Evaluator (PIQE)* (Venkatanath et al., 2015) is another popular metric that differs from the above two metrics in evaluating the quality of an image without using any reference image (No-reference algorithm). This metric instead uses statistical features of an input image to evaluate its image quality. Similar to the SSIM metric, PIQE focuses on human perception-based quality evaluation to measure image quality. This metric inherently provides local measures of quality in addition to global scores. As there are some research studies (Bogatyrev et al., 2023) that have proposed PSNR and SSIM do not sufficiently provide overall quality measures, we have also considered counting PIQE values in the experiments.

The PIQE scores are inversely proportional to image quality where lower scores indicate higher image quality and are computed without any reference image. For consistency, we have complemented the scores with 100 so the high scores represent better quality.

##### 3.3.1.2 Segmentation model performance evaluation

The SSL pretext tasks were evaluated through their performance in downstream segmentation tasks. We employed one of the widely used metrics, the Dice Similarity Coefficient (Dice) score to evaluate the segmentation prediction performance of the downstream task. It is computed as twice the area of overlap between the GT mask and predicted mask over the total area of GT and prediction (see Equation 2 below). We show the scores on a scale of 0 to 100 for consistency across different measures, where a score of 100 indicates a perfect overlap between the predicted mask and GT masks, and a score of 0 indicates no overlap.


(2) $$\begin{array}{l} Dice=2\times \frac{\left(g\cap p\right)}{\left(g+p\right)} \end{array}$$

where *g* is the GT mask and *p* is the predicted mask.

## 4 Results

This section presents the results from the experiments for each dataset including fine-tuned evaluation of the models for segmentation using Dice scores, image quality metrics, and the statistical tests to compare the scores across different SR methods. We also present a graphical user interface we developed to host the models so that domain experts can experiment with them.

### 4.1 Evaluation of SR image quality

**Figure 4:**
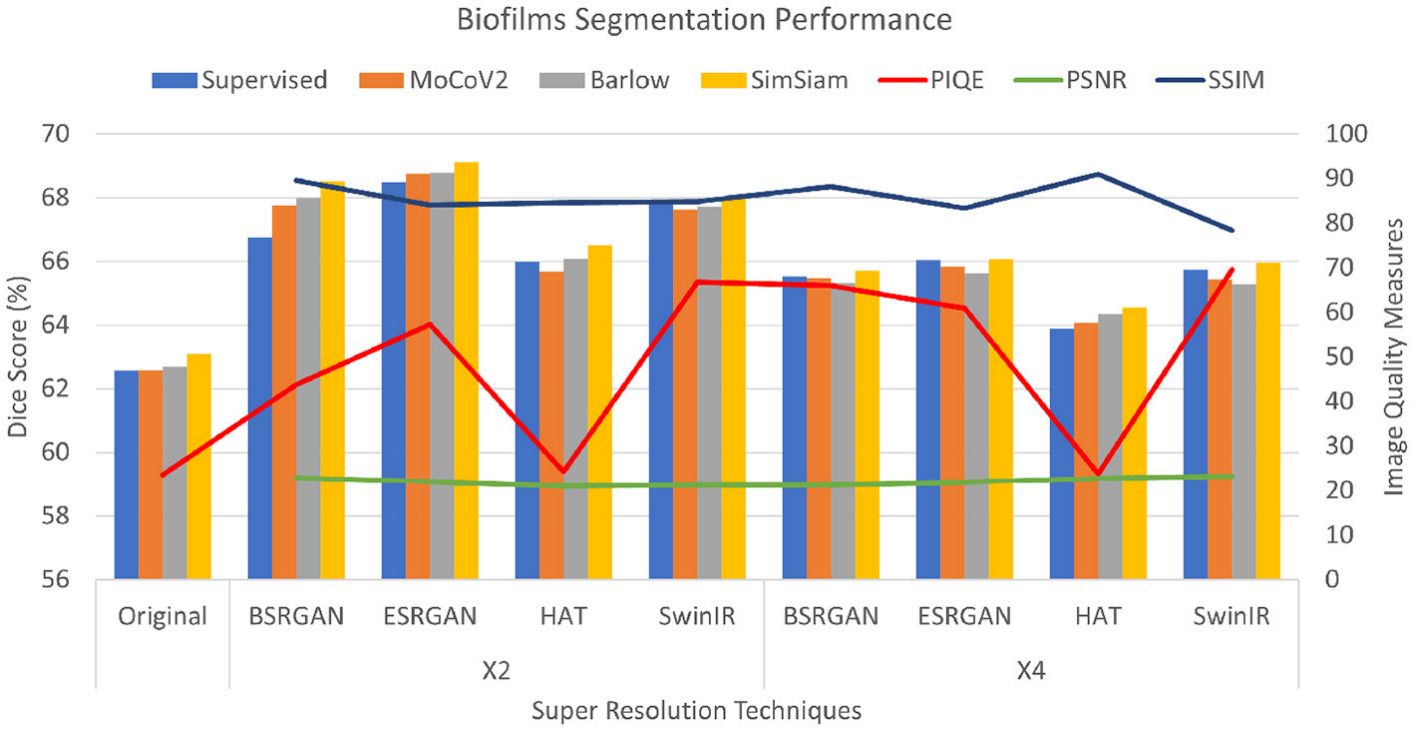
Plots showing Dice scores and Image quality across different SR techniques for Biofilms dataset. The X-axis shows different SR techniques at ×2, ×4 scales along with the original. The Y-axis to the left represents dice scores and to the right represents the Image quality measures for the three metrics.

For the *Biofilm* dataset, the PSNR scores for × 2 and × 4 images in comparison to reference images were consistent between 21 to 22 for all the SR techniques compared to the original LR images. The SSIM scores for × 2 and × 4 images ranged over 0.8, which indicated that the image quality had improved for the × 2 and × 4 images. However, the × 4 images showed slightly lower SSIM scores compared to the × 2 images. The PIQE scores for the models showed a consistent decrease with the original scores being higher than the scores of images at the × 2 scale, which were higher than that of images at the × 4 scale. Consequently, original images showed a lower PIQE score of 23.5, while × 2 images had scores ranging from 39 to 43 with HAT exhibiting scores lower than 30, and the × 4 images score improved further ranging from 60 to 69. This shows improvement in the image quality of × 4 images with scores higher than × 2 images for all the SR techniques. These metrics are shown in Figure 4 as the lines on the bar graph with the right Y-axis showing the measures and the X-axis showing the different SR techniques and × 2 and × 4 scales.

**Figure 5:**
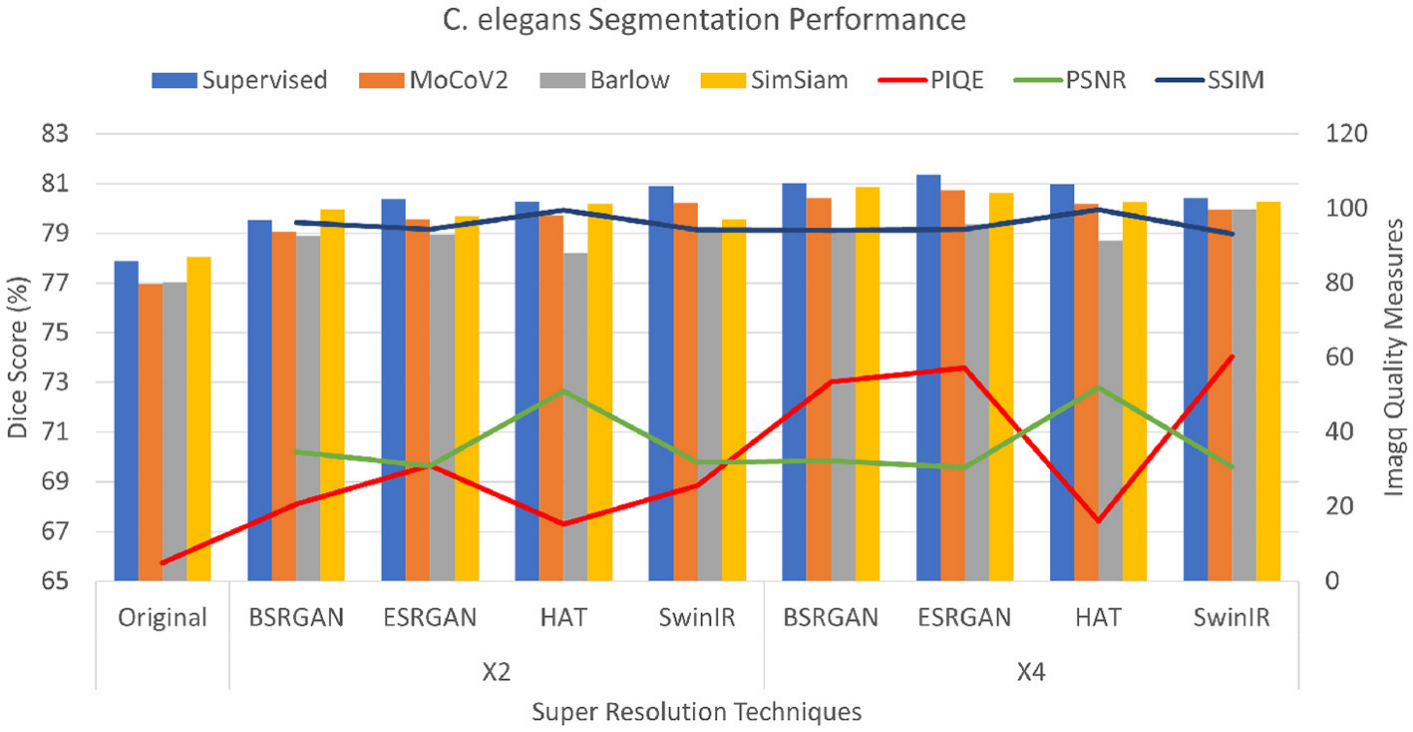
Plots showing Dice scores and Image quality across different SR techniques for C. elegans dataset. The X-axis shows different SR techniques at ×2, ×4 scales along with the original. The Y-axis to the left represents dice scores and to the right represents the Image quality measures for the three metrics.

The *C. elegans* dataset showed PSNR values ranging from 30 to 32 for all the SR techniques but HAT with the scales × 2, and × 4. HAT with × 2 and × 4 scaling showed scores around 50 indicating the best image quality of all. The scores of × 4 images were slightly lower with a variation of 1 to 2 points compared to × 2 images. The SSIM scores were ranging from 93 to 96, demonstrating a significantly higher image quality. Consistently, the complemented PIQE score of the original images was considerably low. The scores for × 2 resolution have increased ranging between 15 and 25 with HAT images showing the lowest scores. The quality of × 4 images showed improved PIQE measures ranging from 53 to 60 with the exception of HAT, showing little or no improvement with × 4 resolution. The metrics are shown in Figure 5.

**Figure 6:**
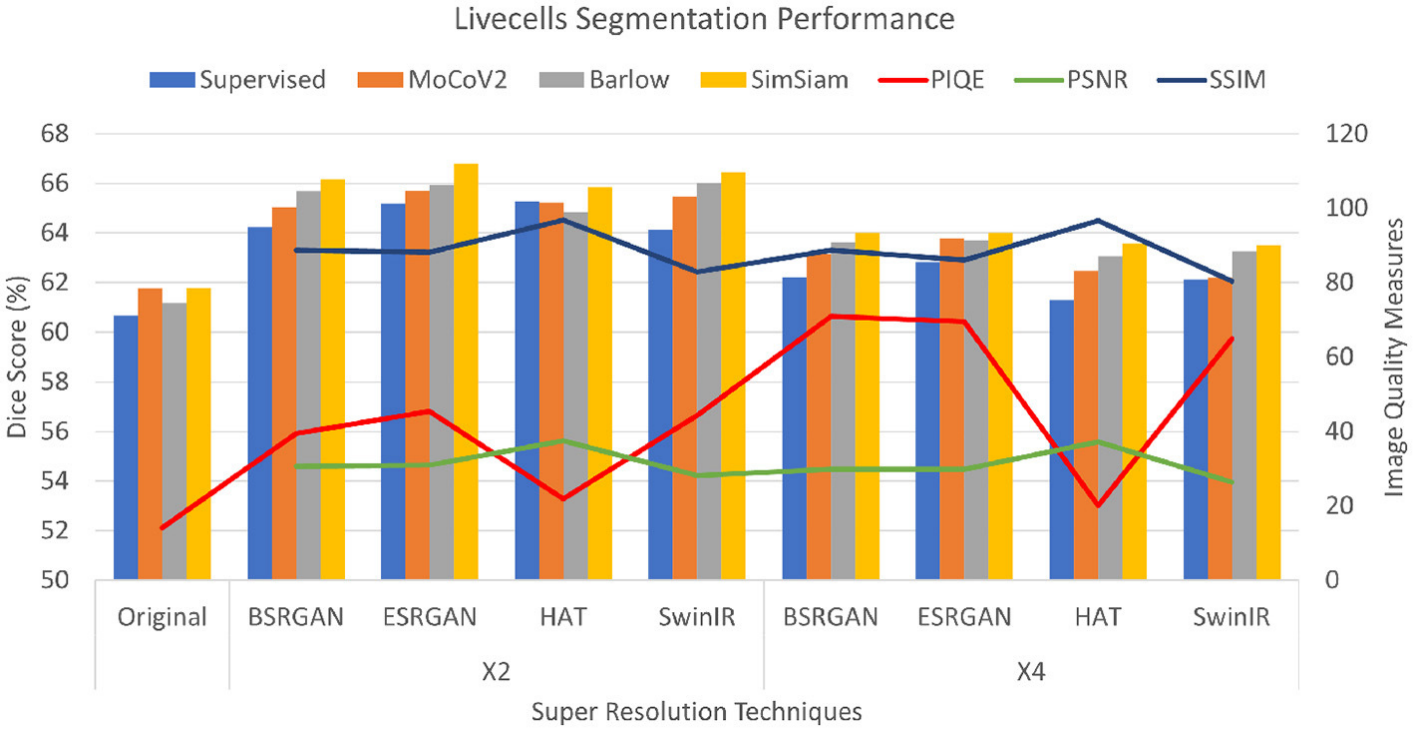
Plots showing Dice scores and Image quality across different SR techniques for Livecells dataset. The X-axis shows different SR techniques at ×2, ×4 scales along with the original. The Y-axis to the left represents dice scores and to the right represents the Image quality measures for the three metrics.

The *Livecells* dataset had PSNR scores ranging from 28 to 37 for × 2 images and × 4 images showed a slight reduction of 1 to 2 points. HAT images had a maximum score of the other images. SSIM images also followed a similar pattern where × 2 images ranged from 82 to 88 with a slight reduction for × 4 images. HAT images showed the highest score of 96. PIQE scores also followed a similar pattern to the other two datasets of consistent increase from original to × 2 to × 4. Original images have a score of 14, × 2 images ranging between 39 to 44 with HAT showing the lowest score of 22. While × 4 images had scored from 64 to 70 with HAT showing no improvement. The metrics are shown in Figure 6.

### 4.2 Evaluation of downstream segmentation task

To compare the effectiveness of using SR images, from various SR techniques, to perform SSL with the respective SR unlabeled images, we chose segmentation as the downstream task. The three representation learning architectures chosen use ResNet50 as their backbone for the encoders. The weights of the network pre-trained on unlabeled data were transferred to FCN ResNet50 for performing segmentation. The segmentation head in FCN Resnet50 was fine-tuned with a few labeled data by freezing the pre-trained weights of Resnet50. For segmentation purposes, we considered segmenting only single class “cells” for each of the datasets.

For the *Biofilm* dataset, the Dice scores for the experiments and their variations across the different resolution techniques with chosen SSL methods for original, × 2, and × 4 images are shown as bar graphs in Figure 4. As the Figure shows, the Dice score of original images in an SL setting was observed to be 62.50±0.016 and the score improved for × 2 resolution highest was observed to be SimSiam trained on × 4 ESRGAN images with the Dice score of 69.37±0.071. Compared to the original images in SL and SSL approaches, the Dice scores of images generated using SR methods improved varying in a range of 2%−6%. The Dice scores of × 4 images slightly dropped compared to that of × 2 images by a range of 1%−2%. Of all the combinations, HAT × 2, and × 4 datasets showed lower performance. The performance of SSL techniques was comparable to that of the SL ImageNet pre-trained setting, at times with SimSiam outperforming the others including the SL approach. MoCoV2 and Barlow Twins showed similar performance.

For the *C. elegans* dataset, the Dice scores for the dataset ranged from 77.01±0.081 to 80±0.062 with original images for the × 2 images. The Dice scores of × 4 images increased by 1%−2% more than × 2 images. SimSiam was observed to show performance comparable to the SL setting. The scores of these experiments are shown as bar graphs in Figure 5.

For the *Livecells* dataset, the Dice score for original images in the SL setting was 60.68±0.001 and ranged around 61±0.072 for the SSL setting. SimSiam outperformed in the experiments with ESRGAN showing the maximum Dice score of 66.70±0.059. × 4 SR images showed slightly lower performance compared to that of × 2 images varying about 1%−3%. The scores are shown in bar charts in the plot in Figure 6.

**Figure 7:**
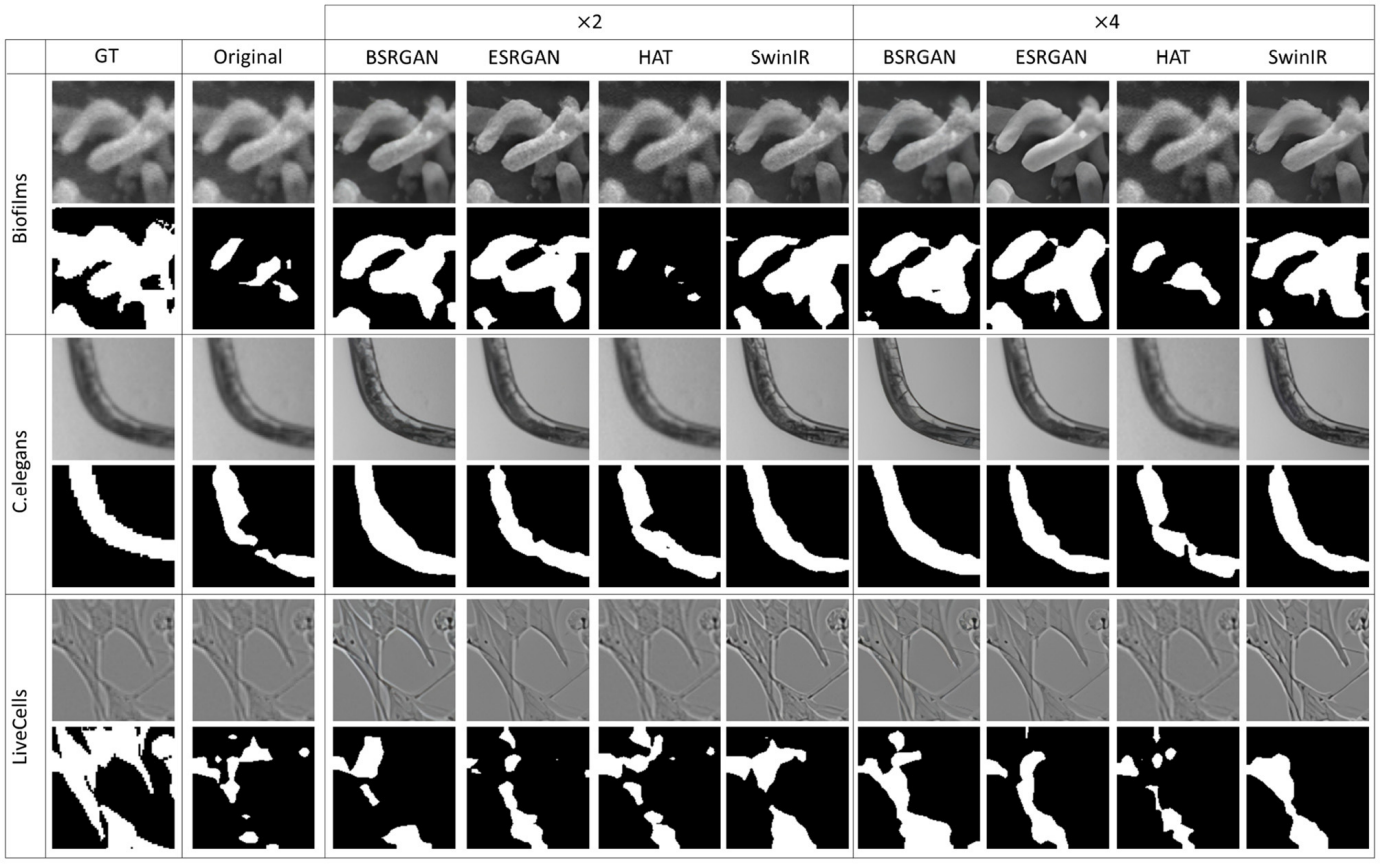
Sample segmentation predictions in the SL setting for the three datasets. The top row inside each dataset block shows the image patches from different SR techniques and scales and the bottom row shows corresponding predicted masks along with the GT mask in the first column.

**Figure 8:**
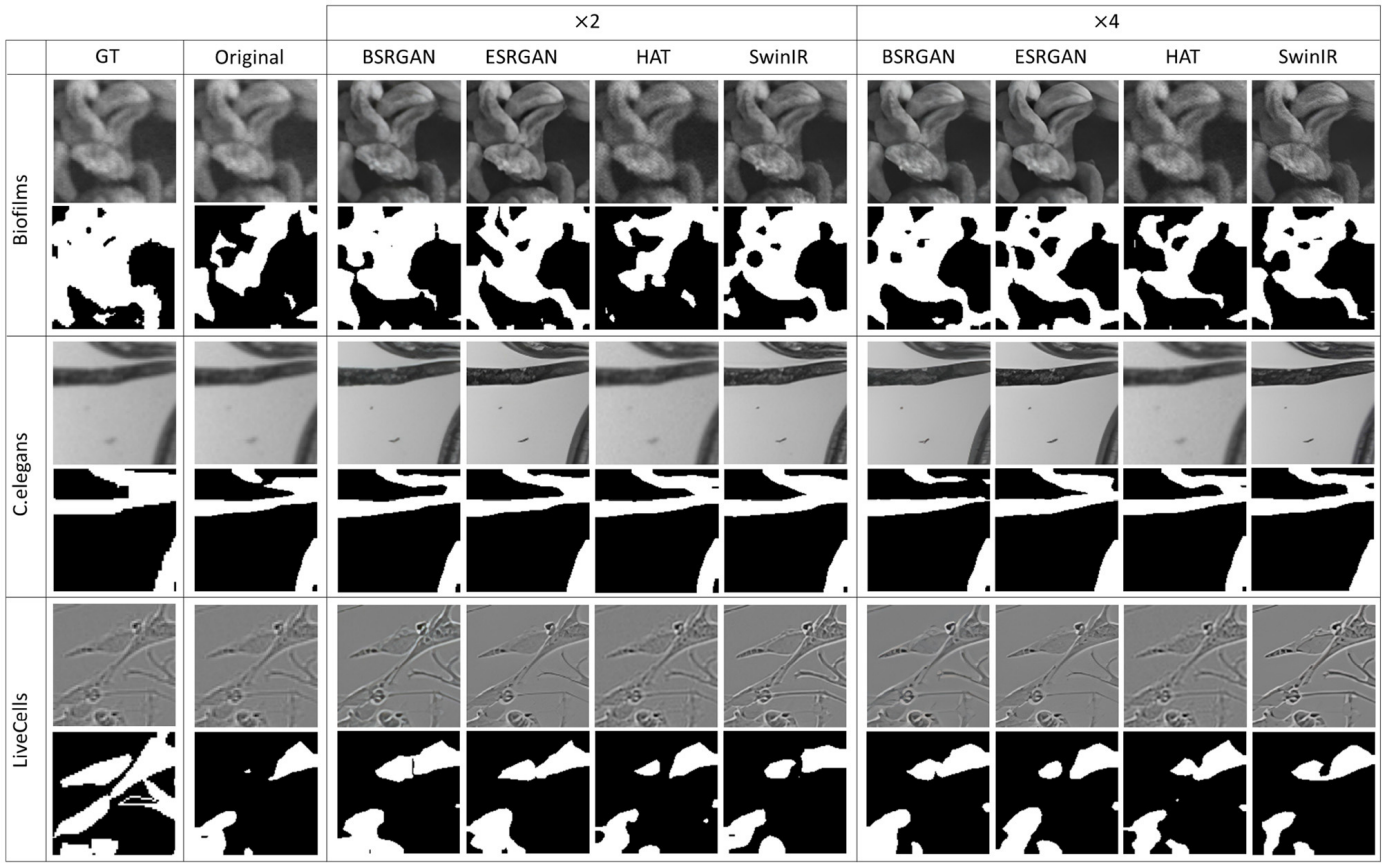
Sample segmentation predictions in SSL SimSiam setting for the three datasets. The top row inside each dataset block shows the image patches from different SR techniques and scales and the bottom row shows corresponding predicted masks along with the GT mask in the first column.

**Figure 9:**
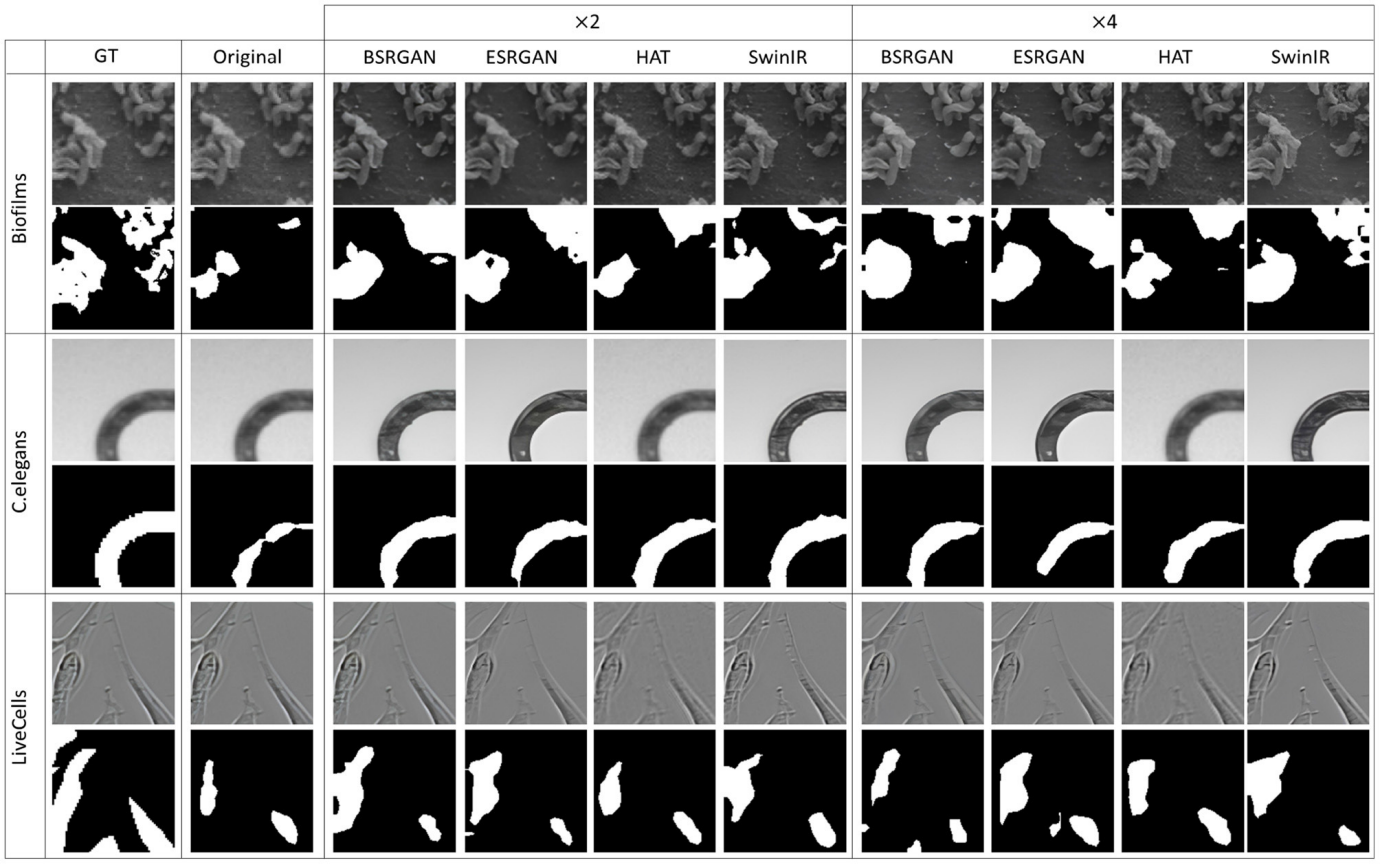
Sample segmentation predictions in SSL Barlow Twins setting for the three datasets. The top row inside each dataset block shows the image patches from different SR techniques and scales and the bottom row shows corresponding predicted masks along with the GT mask in the first column.

**Figure 10:**
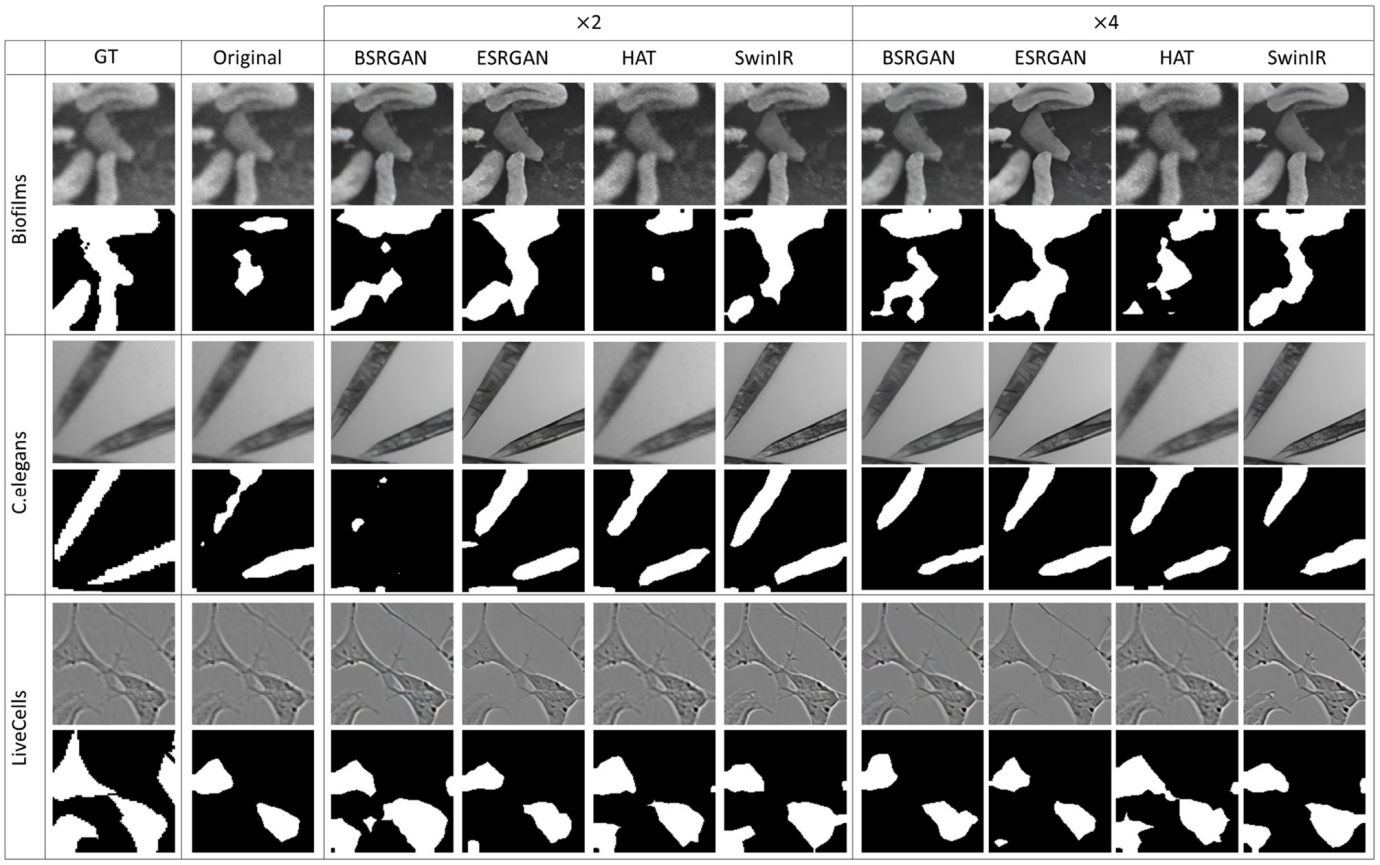
Sample segmentation predictions in SSL MoCoV2 setting for the three datasets. The top row inside each dataset block shows the image patches from different SR techniques and scales and the bottom row shows corresponding predicted masks along with the GT mask in the first column.

Figures 7–10 show image samples and the corresponding predicted segmentation masks for supervised, SimSiam, Barlow Twins and MoCoV2 settings respectively.

### 4.3 Statistical evaluation

For statistical analysis, student t-tests were performed to see if improving image quality improved the segmentation performance by comparing the mean Dice scores. We compared the mean Dice scores of SimSiam and SL settings for all the datasets. The mean Dice scores of original images were compared with all × 2 and × 4 datasets. The mean Dice scores show that for the Biofilms dataset, there was a significant difference between original vs × 2, original vs × 4, and × 2 vs × 4 dataset dice scores (p < 0.001). However, original vs × 4 performances for HAT did not show significant difference. C.elegans dataset did not show a significant difference between mean Dice scores of × 2 vs × 4 scales at α=0.05, however, there was significant difference between original vs × 2 and original vs × 4 scales (p < 0.05). The Livecells dataset of all three different scales showed significant difference in performance (p < 0.001) original vs × 2, original vs × 4, and × 2 vs × 4.

### 4.4 Graphical user interface

**Figure 11:**
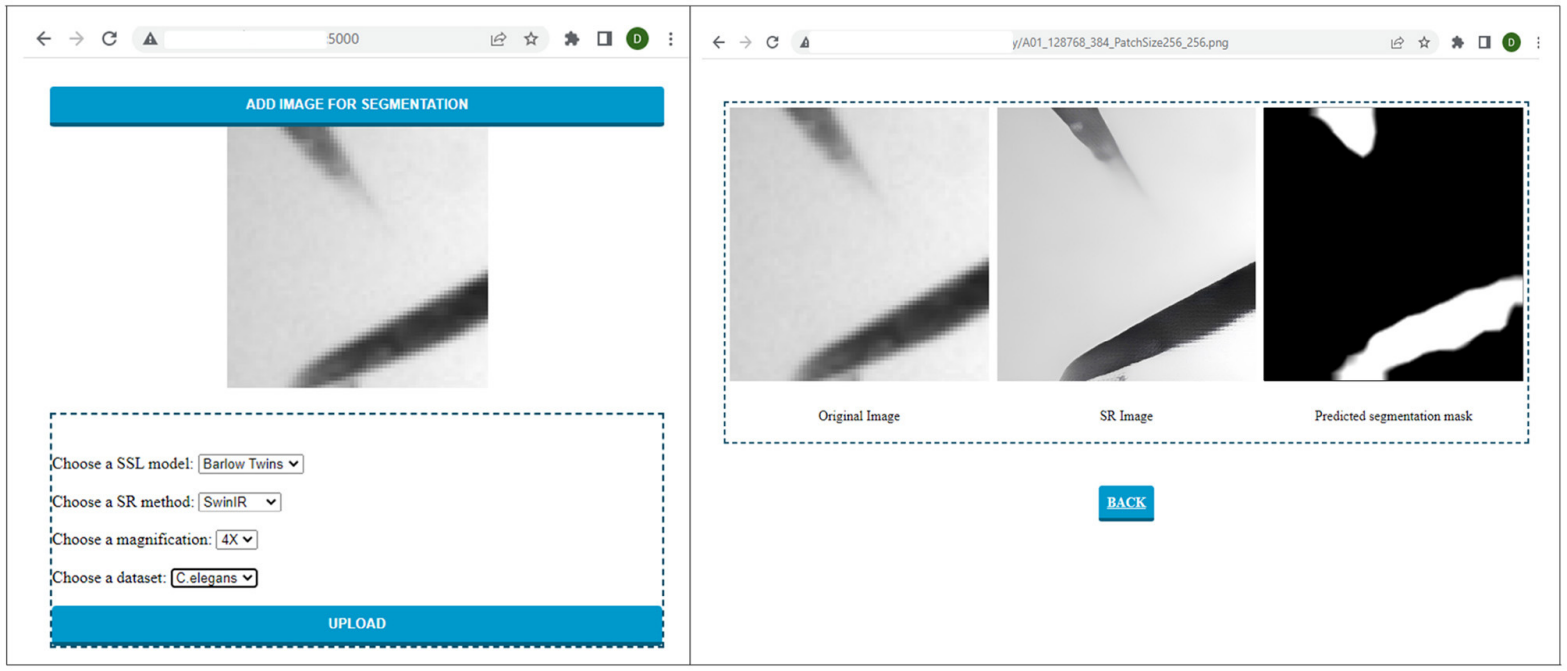
Sample run of the C. elegans image patch on the graphical interface developed to perform and visualize automatic segmentation of low-resolution electron microscopy targets.

To utilize the models trained and experimented with in this paper, we have developed a user-friendly graphical interface as shown in Figure 11. The interface is equipped with all the models trained for segmentation. It enables the user to upload the image patch to be segmented and choose the configuration of choice from the dataset, scale, resolution technique, and SSL technique. After the user selects the original image and the necessary configurations, the result can be automatically displayed to the user. As a result, this system can be used with different datasets in real time by placing the trained models in the backend.

## 5 Discussion

With this study, we aimed to compare how SR of different scales is effective for performing SSL. In this section, we discuss some of the aspects of our experiments and the results.

PSNR and SSIM are reference-based image quality metrics. Recent studies also compared the metrics to show that they are not particularly suitable for assessing the image quality specifically for super-resolution algorithms (Ding et al., 2021; Kirillova. et al., 2022). We observed similar behavior in our experiments that these metrics did not fluctuate over the × 2, × 4 scales for all the SR techniques (see Figures 4–6). However, we observed that non-reference-based metrics, PIQE, were comparatively more representative of the image quality improvement and hence sensitive to the super-resolution scale over reference-based metrics. We then compared PIQE scores to the Dice scores of the segmentation task and observed that PIQE reflects the segmentation model performance.

Based on the experiment results, images with the image quality bound to “*poor”* to “*fair”* (Venkatanath et al., 2015) were found to show better segmentation performance. In the experiments images with × 2 resolution observed the PIQE scores in the range [20, 60], we presume that is one of the reasons for × 2 images exhibiting better performance over original images whose PIQE scores fell in the range [0, 20] or “*bad”* category(see Table 1). However, though PIQE scores for × 4 scale images are higher (60 and above) “*good”* to “*excellent”*, the performance of × 4 images was comparatively lower than × 2 in some scenarios. It can be observed that the PIQE scores and their corresponding Dice scores exhibit similar trends suggesting that the values are correlated. It can be deduced that given a safe range of PIQE “*poor”* to “*fair”*, Dice scores show a near parallel trend. This indicates that the PIQE score can be an important tool for the initial analysis of the images, thereby aiding in further improving the quality using SR techniques accordingly for a desired segmentation performance.

**Table 1:** PIQE quality ranges as used in the experiments.

**Quality scale**	**Score range**
Bad	[0, 19]
Poor	[20, 49]
Fair	[50, 64]
Good	[65, 79]
Excellent	[80, 100]

**Figure 12:**
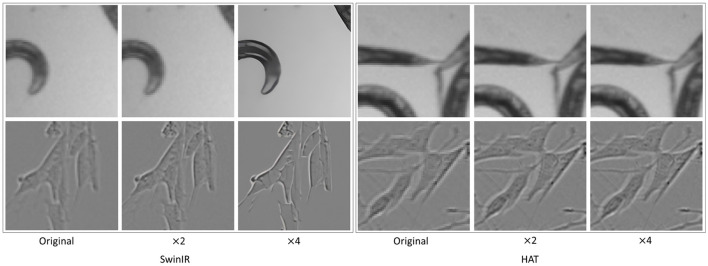
Unintended artifacts introduced by ×4 SR for SwinIR where the image is extra-smoothed. HAT shows little improvement over the original images. The top row shows C. elegans and the bottom row shows Livecells.

No significant differences in segmentation performance were observed between GAN-based and transformer-based SR methods. However, we observed that HAT exhibited a drop in image quality (see Figure 12) and thus the segmentation performance as compared to the other SR techniques. This poor super-resolution performance could occur due to the domain differences between pre-trained data and the test data (HAT SR models trained on natural data). C. elegans dataset showed similar segmentation performances with original, × 2, and × 4. This could be due to the fact that the complexity of features to be learned in this dataset is comparatively lower for the segmentation task. The foreground and background were vivid and highly contrastive even without any resolution enhancements. However, we noticed an interesting behavior of × 4 performances which was slightly better than × 2. We suspect this was due to the × 4 enhancements in terms of texture and intensity contrast (see Figure 12 original, × 2, × 4 images of C. elegans) making the segmentation task better. Further, we have also experimented with another loss function, dice loss, to understand how the performance differs from BCE loss. However, the segmentation results are almost similar to dice and BCE loss functions. We conjecture that BCE performs well because the datasets show balanced ratios of foreground to background pixels, hence BCE was favorable.

Finally, the performance of SSL techniques using only 20% of labeled data showed comparative results to the FL baseline for all the scales. Also, the overall performance of models with × 2 and × 4 scales was improved compared to the original images. Further, with limited labeled data SSL models for higher scales performed as good or better than SL in some scenarios. This shows that SR helps the SSL models to learn representations better from the unlabeled data. Given the scarce availability of labeled images in challenging domains where labeling images is expensive SSL techniques can be a potential substitute to overcome this challenge.

## 6 Conclusion

This study investigates two challenges- the impact of performing SR, as a pre-processing step, on segmentation and leveraging SSL with super-resolved unlabeled images when pixel-wise annotations are limited, specifically for electron microscopy images. For performing SR to improve the quality of images we employed ML-based SR techniques which showed promising results in improving image resolution in various fields. Underlying these challenges, in this empirical study we experimented with various SR techniques on various datasets, compared the effectiveness of SR processing over different SSL methods, and presented the significance over supervised baseline performance. We also studied different metrics used to measure the image quality of SR images and characterized a safe range of SR upscaling in order to achieve optimal electron microscopic image segmentation performance.

The image quality of all the datasets improved significantly with all the SR techniques. However, we observed HAT could not improve the image quality as compared to the other SR techniques and SwinIR introduced some artifacts like extra-smoothing though it improved overall image quality. We observed that of the three metrics PSNR, SSIM, and PIQE, the latter was more representative of the image quality. Also, we observed a dice score improvement of 2%-6% over the supervised baseline with SimSiam performing better than the other SSL models. Another notable observation is that images with × 2 showed better segmentation performance compared to × 4, and × 2 images mostly fall in the complemented PIQE scores in *poor, fair* which is the proposed image quality threshold. Though we observed significant performance improvements, it is essential to conduct further investigation for any potential scenarios where SR techniques negatively impact the model performance. We believe a deeper theoretical explanation of how SR preprocessing improves SSL representation learning would broaden the understanding of the usability of SR preprocessing in electron microscopic image segmentation which could be a potential future direction. Further, it can be speculated that the method would perform better, for instance, segmentation as well. We believe that this would be due to the improvement of feature learning capabilities which would be beneficial for any vision-based applications such as classification, detection, and segmentation. Moreover, image quality improvement and enhanced object boundaries resulting from super-resolution could contribute to other complex vision tasks, such as instance segmentation, which would be another promising area to study.

## Data availability statement

*C.elegans* (Ljosa et al., 2012) and Livecells (Edlund et al., 2021) are publicly available datasets. Any inquiry regarding the private dataset (Biofilms) should be made to the corresponding authors.

## Author contributions

VB, DA, PC, and MS contributed to the conception and design of the study, and wrote the paper. VB and DA implemented the pipeline of models and performed the experiments. PC contributed to Funding acquisition. All authors contributed to manuscript revision, read, and approved the submitted version.
